# Identification and Validation of 17-lncRNA Related to Regulatory T Cell Heterogeneity as a Prognostic Signature for Head and Neck Squamous Cell Carcinoma

**DOI:** 10.3389/fimmu.2021.782216

**Published:** 2021-11-22

**Authors:** Qi Sun, Yumei Li, Xin Yang, Xinxin Wu, Zhen Liu, Yakui Mou, Xicheng Song

**Affiliations:** ^1^ Department of Otorhinolaryngology, Head and Neck Surgery, Yantai Yuhuangding Hospital, Qingdao University, Yantai, China; ^2^ Shandong Provincial Clinical Research Center for Otorhinolaryngologic Diseases, Yantai Yuhuangding Hospital, Yantai, China

**Keywords:** regulatory T cell heterogeneity, long non-coding RNA, prognostic signature, immune cell infiltration, head and neck squamous cell carcinoma

## Abstract

Successful eradication of tumors by the immune system depends on generation of antigen-specific T cells that migrate to tumor sites and kill cancerous cells. However, presence of suppressive Treg populations inside tumor microenvironment hinders effector T cell function and decreases antitumor immunity. In this study we independently evaluated and confirmed prognostic signature of 17-Treg-related-lncRNA. Immune cell infiltration analysis using 17-lncRNA signature as a probe, accurately described Treg populations in tumor immune microenvironment. 17-lncRNA signature model predicted prognosis with excellent accuracy in all three cohorts: training cohort (AUC=0.82), testing cohort (AUC=0.61) and total cohort (AUC=0.72). The Kaplan-Meier analysis confirmed that the overall survival of patients in the low-risk group was significantly better than those in the high-risk group(P<0.001). CIBERSORT analysis confirmed that low risk group had higher infiltration of tumor killer CD8 T cells, memory activated CD4 T cells, follicular helper T cells and T cells regulatory (Tregs), and lower expression of M0 macrophages and Mast cells activated. These results indicate that the 17-lncRNA signature is a novel prognostic and support the use of lncRNA as a stratification tool to help guide the course of treatment and clinical decision making in patients at high risk of HNSCC.

## Introduction

Squamous cell carcinoma of the head and neck is the sixth most common cancer in the world (1). A 5-year survival rate for patients with HNSCC is lower than 50%–55%, mainly due to local recurrence or metastasis (2, 3). The immunotherapy response rate of recurrent or metastatic HNSCC is largely driven by tumor immune microenvironment (TME) and is notoriously poor. A better understanding of TME in HNSCC is critical for the immune therapy to infuse new hope into HNSCC patients. To that end, identification of TME specific biomarkers that can effectively predict efficacy of immune therapy is crucial for proper patient selection.

Tumor infiltrating Tregs which are part of tumor microenvironment are thought to hinder local anti-tumor immune response by mediating tumor immune escape and accelerating its progression (4–6). However, conflicting data regarding poor prognostic value of Tregs raised a possibility of two distinct subtypes present in different tumor types: (I) suppressive Tregs, which are CD4 T cells that express CD45 receptor in both resting and activated states and (II) nonsuppressive Tregs, which are CD45 receptor negative CD4 T cells (7). Infiltration of TME with immunosuppressive Tregs inhibits T cell effector function (8, 9) and NK cell-mediated cytotoxicity (10) leading to poor prognosis and high recurrence of most cancers, including breast and lung cancer. By the same token, presence of non-immunosuppressive Tregs in colorectal cancer (CRC), known to secrete pro-inflammatory cytokines (11), leads to a better prognosis than those infiltrated with suppressive Tregs (12). Mechanistically, nonsuppressive Tregs may benefit the host by inhibiting low levels bacteria-driven inflammation (13).

There are previous reports that have noted that expression of lncRNA correlates with specific Treg subtype expressed during carcinogenesis (14, 15). For example, LINC00301 lncRNA is highly expressed in non-small cell lung cancer. Its expression targets TGF -beta which leads to an increase in a number of immunosuppressive Tregs and a decrease of the CD8T cell positive population in LA-4/SLN-205-derived tumors (15). In hepatocellular carcinoma (HCC), lnc-EGFR (epidermal growth factor receptor) was shown to promote differentiation of immunosuppressive Tregs offering a new therapeutic target for HCC (14, 15). However, which lncRNA specifically regulate Treg cell heterogeneity in HNSCC patients remains unknown.

In this study, we identified a unique 17-lncRNA signature using Univariate Cox and LASSO analysis followed by multivariate Cox regression signature construction. Kaplan-Meier analysis, ROC analyses, and multivariate Cox regression confirmed that THRL signature is indeed a novel, unique and important prognostic factor. 17-lncRNA signature correlated with the infiltration status of Treg cells, and is a true measure of tumor immune microenvironment in HNSCC. Our study will allow for better clinical decision making in evaluating HNSCC patients who will benefit from immunotherapy.

## Methods

### Data Sets and Sample Extraction

RNA sequencing data sets together with the corresponding clinical characteristics of patients with HNSCC were downloaded from The Cancer Genome Atlas (TCGA, https://cancergenome.nih.gov/). Manual reannotation of RNA-seq data sets was used to separate expression data into mRNA and lncRNA. The expression levels were transformed as log_2_(x+1) and standardized (16). Originally, RNA-seq data sets from 546 patients with HNSCC was collected. Only, 453 cases with complete follow-up data which included survival time ≥ 30 days, clinicopathological profile were included in the follow-up analysis.

### Acquisition of Treg-Related mRNA

The GSE15659 dataset, downloaded from the Gene Expression Omnibus (GEO, https://www.ncbi.nlm.nih.gov/geo/) DataSets, contains five T cell subtypes, including 2 suppressive Treg cell subpopulations, and nonsuppressive Treg cell subpopulations, named as Group 1 and Group 2 in our study, respectively. The dataset was based on the GPL570 platform (Affymetrix Human Genome U133 Plus 2.0 Array). Specifically, we reannotated the probe set by the affymetrixHgU133Plus2.0 array and ensured that the probes mapped to the genome were unique. We analyzed the expression differences of transcripts including mRNA and lncRNA between groups 1 and 2 according to the standard of | log2FC | > 1 and P value < 0.05. The differential mRNAs in GSE15659 were crossed with mRNAs obtained from the expression profiles of patients with HNSCC from the TCGA database, and the crossed mRNAs were listed as Treg-related mRNAs.

### Identification of Treg-Related lncRNA

All patients with HNSCC were randomly divided into training and testing cohorts according to their survival status at the ratio of 1:1 (16). In the training cohort, the correlation score between lncRNAs obtained from TCGA data set and Treg-related mRNAs were calculated by Person correlation coefficient test. LncRNAs that correlated with Treg-related mRNAs were selected for subsequent analysis according to the following criteria of | correlation coefficient | > 0.6 and P < 0.001 (17–19). Finally, a Treg-related mRNAs-lncRNAs co-expression network was constructed according to the criteria of | correlation coefficient | > 0.3 and P < 0.001 (20).

### Identification of Treg-Related lncRNA Signature

To identify survival-related LncRNAs among Treg-related LncRNAs, we performed univariate Cox proportional hazard analysis (21). Treg-related LncRNAs in univariate analysis P<0.01 were included in the Least Absolute Shrinkage and Selection Operator (Lasso) regression. A multivariate Cox regression model was finally used to construct a prognostic signature based on the candidate Treg-related lncRNAs generated from the Lasso regression results (22–24).

### Construction and Application of Prognostic Model

A multivariate Cox regression model was used to establish an independent prognostic signature. The risk score for each patient sample was calculated as the expression value of each lncRNA multiplied by the sum of their weights in the multivariable Cox model. The median risk score was used to separate patients into high- and low-risk groups. To validate the predictive value of the model, we performed the Kaplan–Meier log-rank test and time-dependent ROC curve analysis which were used to compare survival between high and low-risk groups in the training, testing, and total cohorts.

### Validation of Prognostic Signature

Univariate and multivariate Cox regression analyses were performed on the clinical data to determine whether the risk score is an independent indicator of prognosis. ROC curve analysis by univariate and multivariate Cox regression was performed to analyze the correlation between prognosis and clinicopathological factors including age, gender, tumor size (T), lymph node metastasis (N), distant metastasis (M), clinical stage, and risk score. Time-dependent receiver operating characteristic (ROC) curves were plotted to evaluate the accuracy of different clinicopathological factors and risk scores in predicting survival time by using the survival ROC R package (25). Finally, we constructed a prediction model by using nomogram. We then tested the accuracy of the prediction model through 3-year and 5-year calibration curves.

### Correlation Analysis of the 17 Treg-Related lncRNAs With Clinicopathological Factors

The expression of the 17 Treg-related lncRNAs were correlated with clinicopathological factors using the ggpubr R package. Moreover, we analyzed the correlation of 17 Treg-related lncRNAs the overall HNSCC patient survival.

### Analysis of Tumor Immune Microenvironment in High- and Low-Risk Patient Group

Principal component analysis (PCA) was used for effective dimensionality reduction, pattern recognition, and exploratory visualization of high-dimensional data of the whole-genome expression profiles retrieved from TCGA, 462 Treg-related coding genes, and 17-lncRNA signature expression profiles. Gene set enrichment analysis of (GSEA, http://www.gsea-msigdb.org/gsea/index.jsp) in high- and low-risk groups. GSEA was used for gene functional annotation and is a powerful analytical method for comparing genes with predefined gene sets obtained from whole genome expression profiles (26). In our experience, GSEA tends to have high repeatability and explanatory power in the analysis of molecular map data. Finally. gene expression matrix data were screened and analyzed by CIBERSORT (https://cibersortx.stanford.edu/) (27). Specifically, immune cell populations of infiltrating T cells in high- and low-risk groups were compared to access the relationship between 17-lncRNA signature and immune cell infiltration.

## Results

### Identification of Treg-Related mRNA

**Figure 1:**
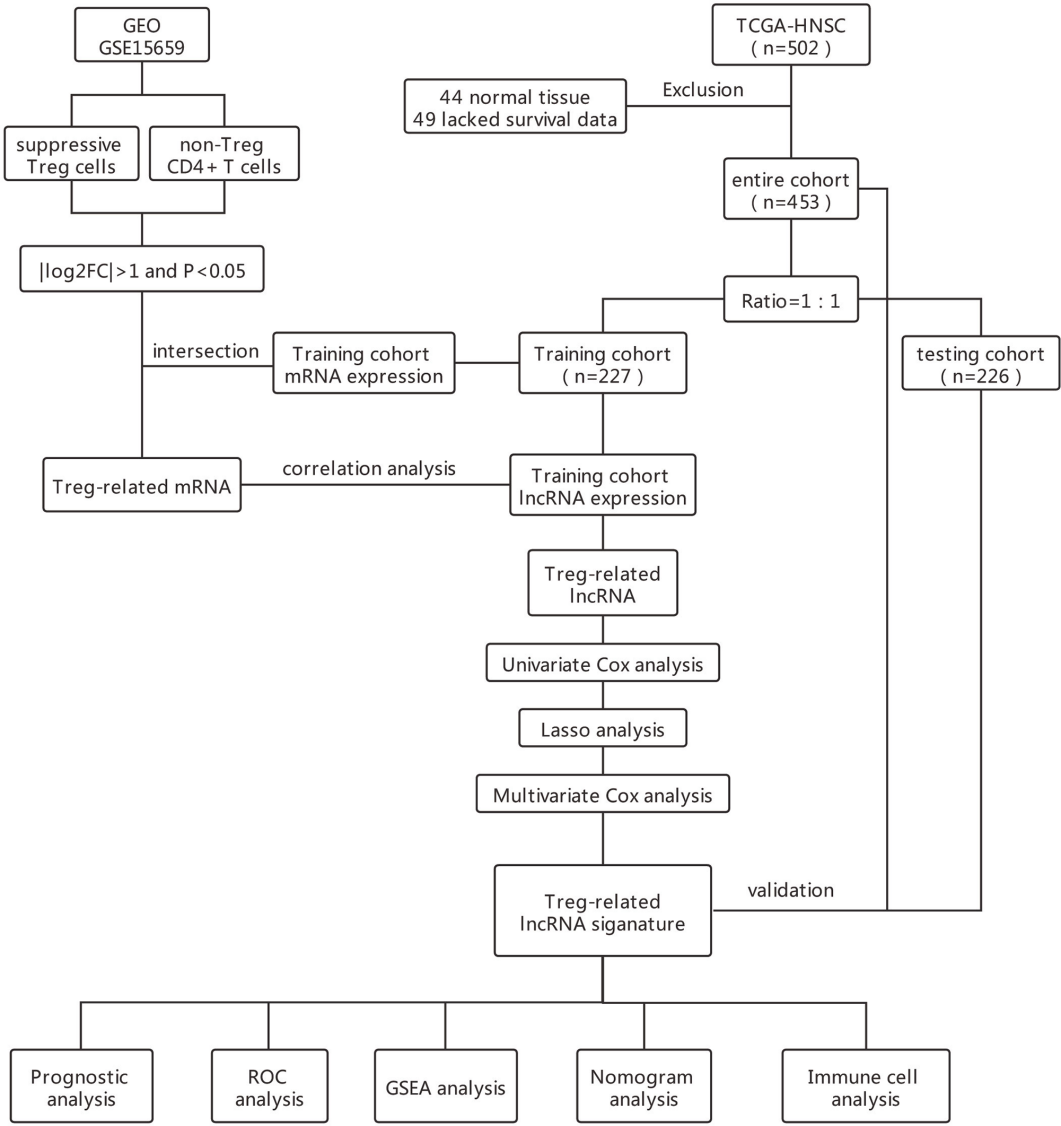
Flowchart of the study.

**Figure 2:**
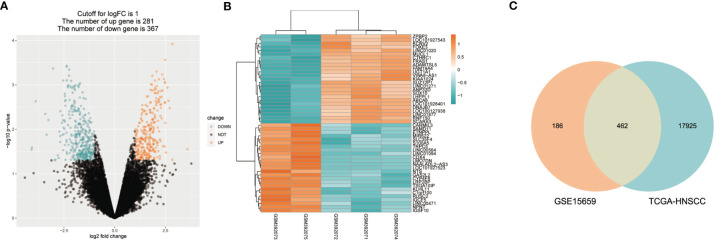
Treg-related mRNA extraction from RNA expression profiles. **(A)** GSE15659 differential gene expression volcano map, screening criteria I log2FC I > 1 and P < 0.05. ln blue are down-regulated transcripts and in red are up-regulat­ ed transcripts. **(B)** Top 50 differential transcripts extracted from GSE15659 shown using heatmap, where blue represents down-regulated transcripts and red up-regulated transcripts. **(C)** Venn Diagram of 462 overlapped mRNAs found in 648 differential expression transcripts from GSE15659 and 18392 mRNA from TCGA-HNSCC.

The flowchart in **Figure 1** describes the order of computational steps we undertook to identify 17 lncRNAs. First, we obtained 18,392 mRNA and 9,357 lncRNA expression profiles which corresponded to clinical data from 453 patients registered in TCGA database. In the GSE15659 dataset from GEO, a total of 648 differentially expressed transcripts were obtained (**Table S1**). The data was divided into 281 up-regulated and 367 down-regulated transcripts and visualized using Volcano plot (**Figure 2A**). The top 50 differentially expressed transcripts were graphed as heat map using the heatmap R package (**Figure 2B**). 462 mRNA present in both GSE15659 and TCGA datasets were identified using Venn Software Analysis. The resulting overlapping mRNA were named Treg-related mRNAs for HNSCC (**Figure 2C**). Data enrichment was used to gather functional information (GO and KEGG) utilizing the Database for Annotation, visualization and integrated discovery (DAVID). Biological pathways related to Treg cell expression, included insulin secretion pathway (28, 29), pancreatic secretion pathway, Hippo signaling pathway (30–32) and others (**Figure S1A** and **Table S2**). In addition, the Treg-related mRNA transcripts related to microglial cell activation, cell adhesion and cell-cell communication were detected (**Figure S1B** and **Table S2**).

### Identification of 17-Treg-Related-lncRNA Signature for the Prognosis of Patients With HNSCC

**Figure 3:**
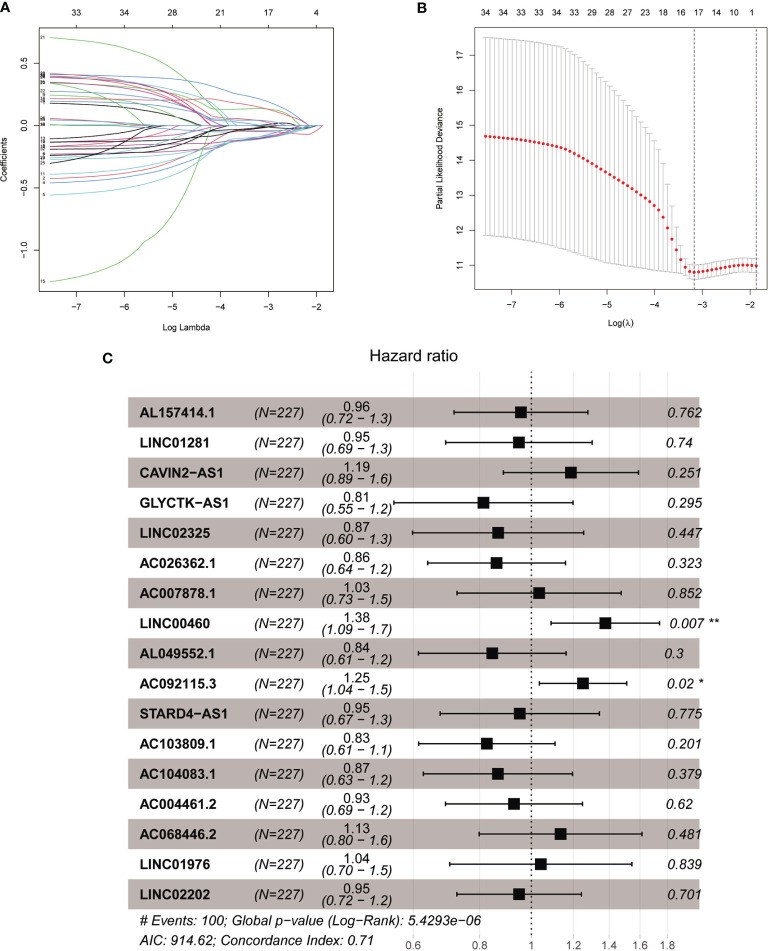
The acquisition of Treg-related lncRNA signature. **(A)** Lasso coefficient distribution of 35 lncRNAs in the train­ ing cohort. **(B)** The coefficient profile is generated according to the logarithmic λ sequence. Selection of optimal parameter λ in lasso model. **(C)** Lasso regression analysis screened forest maps of 17 candidate Treg-related lncRNA related to HNSCC survival and the construction of prognostic signature. *P < 0.05, **P < 0.01.

The total cohort of 453 patients with HNSCC was randomly divided into training and testing cohorts at ratio of 1:1. In the training cohort, 1652 lncRNAs correlated with Treg-related mRNAs in patients with HNSCC (**Table S3**) and were designated as Treg-related lncRNAs. Following that, 35 lncRNAs were identified by Univariate Cox regression analysis (**Table S4**). Lasso regression analysis was carried out on 35 prognostic lncRNAs to improve confidence in the prediction. We identified 17 lncRNAs prognostic factors for patients with HNSCC (**Figures 3A, B**) (22). The correlation between Treg-related mRNAs and the associated lncRNAs is shown in **Figure S2** and **Table S5**. Of 17 assigned lncRNAs, additional multivariate Cox regression analysis of defined transcripts LINC00460 and AC092115.3 as significant independent prognostic factors (**Figure 3C**). These lncRNAs were then used to establish lncRNA prognostic signature. Six lncRNAs, namely, CAVIN2-AS1, AC007878.1, LINC00460, AC092115.3, AC068446.2, and LINC01976, were used to calculate risk factors for the prognosis of patients with HNSCC using the cutoff of HR>1. Meanwhile, 11 lncRNAs (AL157414.1, LINC01281, GLYCTK-AS1, LINC02325, AC026362.1, AL049552.1, STARD4-AS1, AC103809.1, AC104083.1, AC004461.2, and LINC02202) were protective factors for the prognosis of patients with HNSCC with the cutoff of HR<1 (**Figure 3C** and **Table S6**). The risk score for prognosis was calculated as $\text{Risk score}=\Sigma_{i}Expi(lncRNA_{i})*Coef(lncRNA_{i})$ where Expi is the expression value of each lncRNA, and Coef is the regression coefficient of the multivariate Cox analysis for the target lncRNA (21, 23).

### Validation of the 17 -lncRNA Signature for HNSCC Prognosis

**Figure 4:**
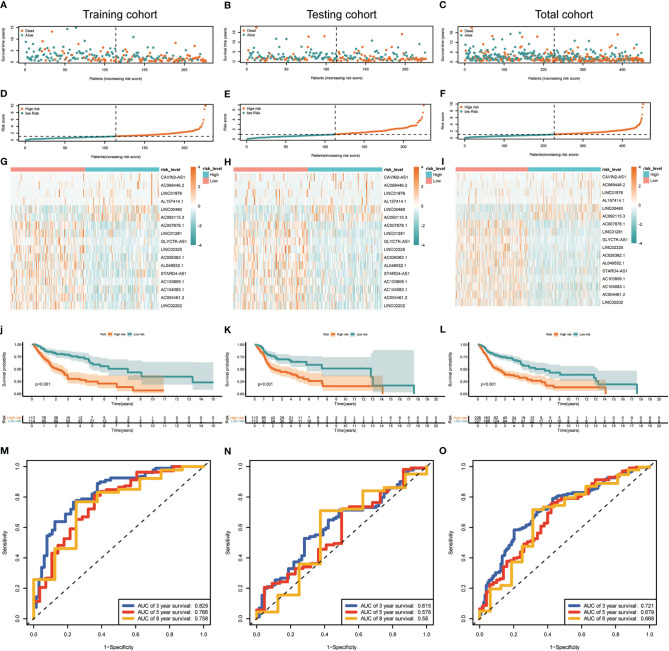
Construction and evaluation of the 17-lncRNA signature. **(A–F)** 17-lncRNA signature risk score analysis. The distribution of the scatter chart of the sample survival overview in **(A)** the training cohort, **(B)** the testing cohort and **(C)** total cohort. The distribution of risk scores in **(D)** training cohort, **(E)** testing cohort and **(F)** total cohort. Green dots and red dots denote survival and death, respectively. **(G–I)** The heat map of the expression profile distribution of 17-lncRNA signature among the low-risk group and high-risk group in the **(G)** training cohort **(H)** testing cohort and **(I)** total cohort. The pink bar represents low-risk group and the blue bar indicates high-risk group. **(J–L)** Verification of Treg-related lncRNA prognostic signature. The risk score level of the model-based classifier, Kaplan-Meier survival analysis was used to analyze the risk of death in the **(J)** training cohort, **(K)** testing cohort and **(L)** total cohort of HNSC's overall survival curve. **(M–O)** Time-depen­ dent receiver operating characteristic (ROC) analysis of the sensitivity and specificity of the survival for the 17-lncRNA signature risk score in **(M)** training cohorts, **(N)** testing cohorts and **(O)** total cohorts.

Median risk score for 17-lncRNA obtained in prognosis model was used to divide patients with HNSCC into high- and low-risk groups. We first used scatter plot (**Figures 4A–C**) and risk curve (**Figures 4D–F**) to describe the risk score and survival status of each patient with HNSCC, in the training, testing and total cohorts. The risk coefficient and mortality rate in the low-risk group were lower than those in the high-risk group. The observed mortality rate correlated with the risk score (**Figures 4A–F**). The heatmap showed that CAVIN2-AS1, AC068446.2, LINC01976, AL157414.1, LINC00460, and AC092115.3 were highly expressed in the high-risk group, while AC007878.1, LINC01281, GLYCTK-AS1, LINC02325, AC026362.1, AL049552.1, STARD4-AS1, AC103809.1, AC104083.1, AC004461.2, and LINC02202 were highly expressed in the low-risk group (**Figures 4G–I**). To assess further the accuracy of 17-lncRNA prognostic signature, we constructed the K-M survival curve using the R package “survival”. In the training cohort, the overall survival (OS) of the low-risk group was better than that of the high-risk group (P < 0.001, **Figure 4J**). These results in the testing cohort (P < 0.001, **Figure 4K**) and total cohort (P < 0.001, **Figure 4L**) were consistent with the results of the training cohort. The risk score correlated with the OS time of patients with HNSCC and hence is a good predictive value for HNSCC prognosis. To further evaluate model quality, we calculated area under the Curve (AUC) for the 3, 5 and 8-year survival curves using the time ROC R package. The AUC values were 0.829, 0.766, and 0.758 in the 3-, 5-, and 8-year follow-ups of the training cohort (**Figure 4M**) and 0.615, 0.578, and 0.58 in the testing cohort (**Figure 4N**). In the total cohort, the values were 0.721, 0.679, and 0.668, respectively (**Figure 4O**). Hence, the 17-lncRNA signature is a reliable measure of prognostic signature of HNSCC.

### Risk Score Is Independent Prognostic Factor for Patients With HNSCC

**Figure 5:**
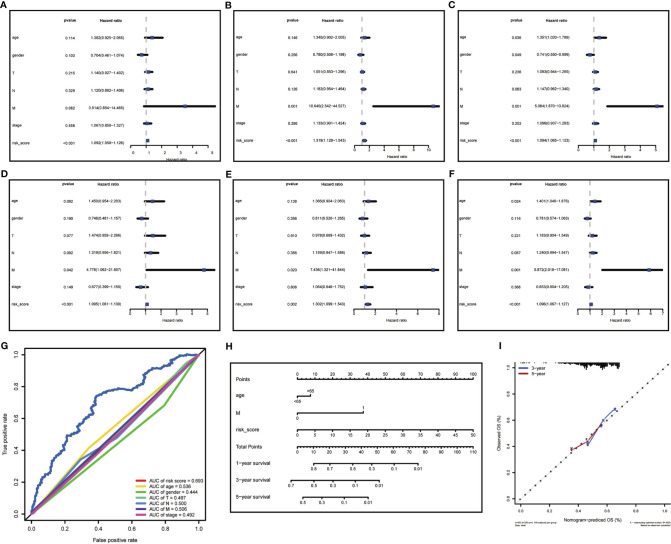
Risk score analysis and nomogram construction to evaluate of overall survival in of HNSCC patients. **(A–C)** The independent prognostic value of risk score was evaluated by Cox regression analysis. Univariate Cox regression analysis of models in **(A)** training cohort, **(B)** testing cohort and **(C)** total cohort. **(D–F)** The independent prognostic value of risk score was evaluated by Cox regression analysis. Multivariate Cox regression analysis of models in **(D)** training cohort, **(E)** testing cohort and **(F)** total cohort. **(G)** ROC analysis of the ability of risk score and other clinicopathological factors to predict the overall survival of HNSCC. **(H)** Nomogram for predicting the overall survival rate of HNSCC. **(I)** Nomogram calibration chart during 3-year and 5-year follow-up.

To further evaluated the risk score as an independent prognostic marker for patients with HNSCC, we performed univariate COX regression analysis comparing the risk score to other clinicopathological factors (age, gender, tumor size (T), lymph node metastasis (N), distant metastasis (M), clinical stage) using the survival ROC R package (**Figures 5A–C**). Age, gender, M, and risk score were independent prognostic indicators for patients with HNSCC. Through the multivariate COX regression analysis, we found that M and risk score were correlated with OS in the training cohort (**Figure 5D**). The multivariate COX regression analysis result of ‘M’ for OS showed p values <0.05 in the training, testing (**Figure 5E**) and total cohorts (**Figure 5F**), indicating that M was a significantly independent prognostic factor for HNSCC. In the same way, ‘risk score’ was also a significantly independent prognostic factor for HNSCC in the training, testing, and total cohorts. The multivariate COX regression analysis suggested that the risk score containing 17-lncRNA signature was a significant prognostic factor for HNSCC, independent of clinicopathological parameters. The sensitivity and specificity of the risk score in predicting the prognosis of patients with HNSCC was investigated by comparing the area under the ROC curve (**Figure 5G**) which measured changes in the risk score and other clinicopathological factors in predicting the overall survival of HNSCC patients. The AUC values of the risk score, age and M were 0.693, 0.536, and 0.506, respectively. The risk score showed a better AUC than other clinicopathological factors, indicating that risk score is more effective in predicting HNSCC prognosis.

In attempt to develop a clinically applicable method for predicting the survival probability of patients, we generated a nomogram using the rms R package (**Figure 5H**), plotting risk score, age, and M. The risk score had the biggest contribution to the 3- and 5-year OS of patients with HNSCC. In addition, we supplemented our model with the 3-year and 5-year calibration charts. The 3-and 5-year OS calibration curves were well predicted compared with the ideal models in all cohorts (**Figure 5I**). The results showed that the nomogram can independently evaluate survival of patients with HNSCC, which may help doctors to make better medical decisions and follow-up plans.

### Correlation of the Expression of the 17-lncRNA Signature With Clinicopathological Factors

The 17-lncRNA signature has been revealed effective for predicting HNSCC prognosis in aforementioned algorithm. However, whether each lncRNA has correlation with clinicopathological factors still remains unclear and needs to be further assessed separately (**Figure S3**). Eleven lncRNAs were associated with disease progression, and included AL157414.1, LINC01281, CAVIN2-AS1, GLYCTK-AS1, LINC02325, AC026362.1, AC007878.1, LINC00460, AL049552.1, AC092115.3, STARD4-AS1, AC103809.1, AC104083.1, AC004461.2, AC068446.2, and LINC01976. Furthermore, three lncRNAs, namely, AC092115, GLYCTK-AS1, and LINC02202, were associated with different TNM classification of HNSCC. The expression of six lncRNAs such as LINC02325, AC026362.1, AL049552.1, AC103809.1, LINC01976, and LINC02202 was statistically significant between different genders, while no statistically significant difference was observed for 17 lncRNAs among different age groups (**Figure S4**). Kaplan-Meier curves reveal that high expression of LINC00460, AC092115.3 and low expression of LINC01281, LINC02325, AC026362.1, AC007878.1, AL049552.1, STARD4-AS1, AC104083.1, AC004461.2 was associated with poor survival (P<0.05). Only ten of the 17 individual lncRNAs had a survival prediction power for HNSCC.

### Differences of Tumor Immune Microenvironment Between the Low- and High-Risk HNSCC Groups

**Figure 6:**
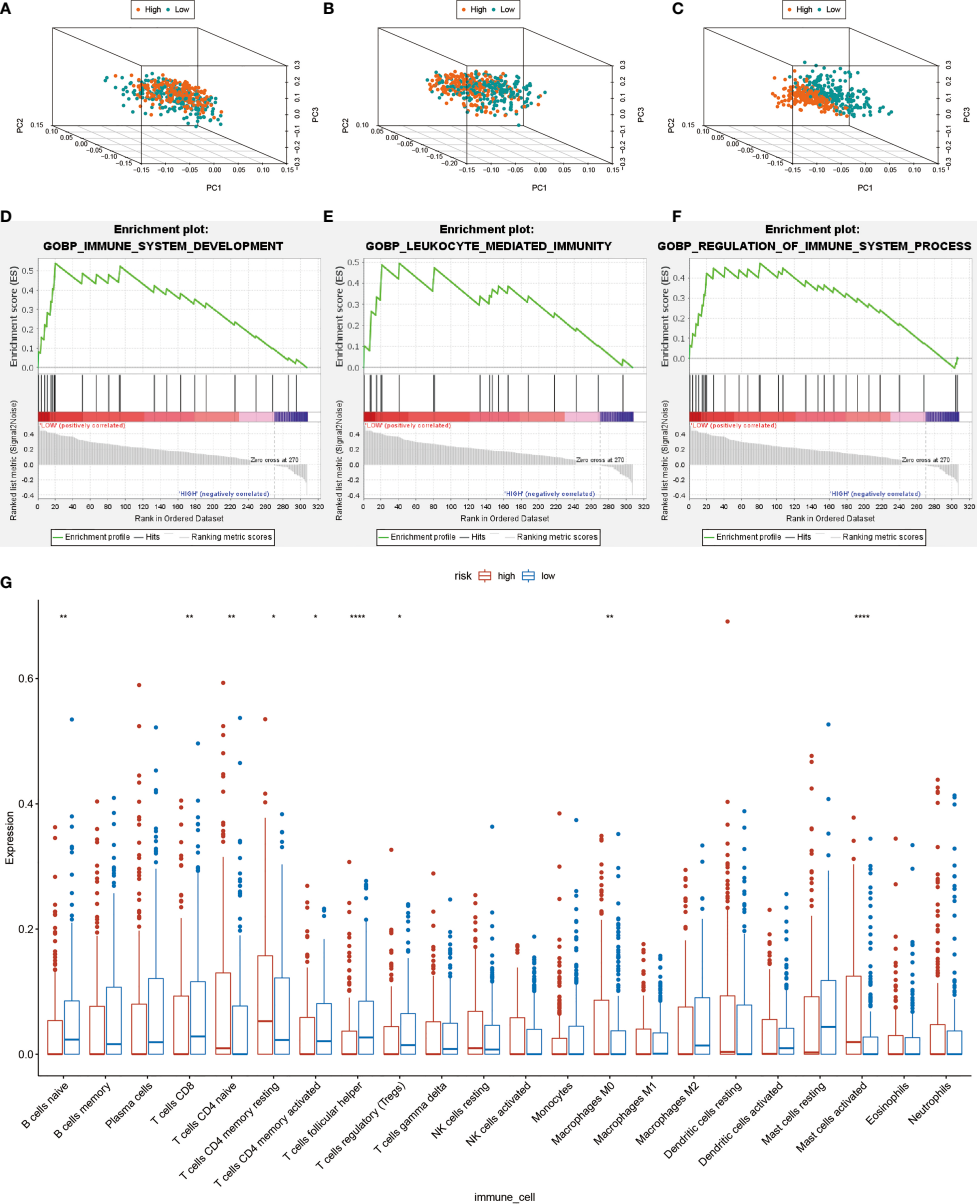
Computational analysis of immune cell infiltration in HNSCC patients. **(A)** All cohort PCA diagrams of genome-wide expression profiles of TCGA. **(B)** Cohort PCA diagrams of 462 Treg-related mRNA. **(C)** PCA diagram of all cohort of 17- lncRNA signature. **(D–F)** GSEA analysis. Results of functional enrichment of GSEA genes in different groups. **(G)** the difference in the expression of infiltrating immune cells between the high-risk group and the low-risk group. *P < 0.05, **P < 0.01, ***P < 0.001, ****P < 0.0001.

Principal Component Analysis (PCA) was performed to evaluate expression differences among the low- and high-risk groups. Whole-genome expression profiles from the TCGA and 462 Treg-related genes was insufficient to separated high and low risk groups (**Figures 6A, B**). However, the expression differences in 17-lncRNA signature could obviously distinguish high-risk patients from low-risk patients (**Figure 6C**). The GSEA analysis confirmed that mRNAs related 17-lncRNA detected in the low-risk groups were enriched for transcripts related to immune-related biological processes- immune system development, leukocyte mediated immunity, and regulation of immune system process (**Figures 6D–F**). and in addition, the low-risk group had a better overall immune response than the high-risk group. The infiltrating immune cells in the HNSCC microenvironment were analyzed by CIBERSORT to explore the differences of immune cell infiltration between high-and low-risk group. Genes related to the naive B cells, CD8 T cells, memory activated CD4 T cells, follicular helper T cells, and regulatory T cells (Tregs) were found to be expressed at higher levels in low-risk group rather than in high-risk group (**Figure 6G**). In addition, expression of naive T cells, memory resting CD4 T cells, macrophage M0, and activated mast cells was significantly lower in low-risk group than in high-risk group (**Figure 6G**). These results suggest that 17-lncRNA signature model is capable of distinguishing tumor microenvironment in the low and high-risk groups of HNSCC patient population.

## Discussion

A subgroup of CD4+T cells, referred to as Treg cells can inhibit anti-tumor immune response and mediate tumor immune escape (33). Therapeutic targeting of Treg cells can effectively prolong the survival time of patients with tumors. Although Treg cell infiltration plays a central role in the pathogenesis of cancer, the heterogeneity of Treg cells paints a conflicting picture as to the ultimate effect of Tregs on cancer progression and patient prognosis. Suppressive Treg cells inhibit immune response leading to immune escape of tumors (33). Activated Tregs, on the other hand, mediate tumor suppression by secreting immunosuppressive cytokines such as IL-10 (34). The resting Tregs, expressing lower levels of FoxP3, mediate suppression by secreting immunosuppressive cytokines such as TGF-β and are less potent at suppressing proliferation and cytokine production by effector T cells (7, 34). Non-suppressive Treg cells are known to secrete pro-inflammatory cytokines and their presence was correlated with tumor invasion by bacteria (7, 12). In the TME, lncRNAs regulate expression of molecules (e.g. PD-L1, MHC I, and HLA-G) on the surface of the tumor cells, which may help attenuate the function of effector T cell (35).

The goal of this study was to identify and validate lncRNA prognostic signature that gives rise to regulatory T cells, which can help provide a more individualized risk-assessment for HNSCC treatment. The prognostic value of 17-lncRNA was confirmed and validated by rigorous computational techniques including Kaplan-Meier analysis, ROC analyses, and multivariate Cox regression. We learned that the 17-lncRNA signature could accurately reflect the infiltration status of Treg cell, hence permitting identification of high-risk HNSCC patients with poor survival outcomes.

Among previously reported 17 lncRNAs, only LINC00460, LINC01281, STARD4-AS1, and LINC02202 were studied in HNSCC. Jiang (36) reported that LINC00460 could effectively induce epithelial-mesenchymal transition in Peroxiredoxin-1 dependent manner, enhancing the tumor cell proliferation and metastasis of HNSCC. LINC00460 was also shown to reduce stanniocalcin-2 by up-regulating microRNA-206 which triggers cellular autophagy, thereby affecting the progression of HNSCC (37). The molecular mechanism, by which LINC00460 influences the tumor microenvironment and regulates Treg cells remains unknown.

LINC01281 were reported to modulate the overall survival rate of patients with laryngeal squamous cell carcinoma by activating Wnt signaling pathway important for cellular proliferation, migration, and apoptosis (38). Our analysis also identifies LINC01281 as a potential biomarker for patients with HNSCC. Fourteen additional lncRNAs have not been previously reported to be potential prognostic targets for HNSCC. lncRNA AC092115.3 is a new novel signature that significantly associated with the prognosis of patients with HNSCC. We also found a high positive correlation between AC092115.3 and pirin (PIR), which can interact with oncoprotein B-cell lymphoma 3 code and nuclear factor I and participate in the activation of nuclear factor κB (NF-κB) (39, 40). Classical NF-κB subunits p65 and c-Rel play a key role in the identity and function of Tregs (41, 42). Hence, AC092115.3 may affect the inhibitory effect of Treg cells on tumor immunity by regulating NF-κB transcriptional activity of specific target genes mediated by PIR.

Many additional differences in immune cell infiltration between high- and low-risk patients with HNSCC were discovered using 17-lncRNA signature model. For example, the infiltration levels with anti-tumor T cells like CD8 T cells, CD4 memory activated T cells, and follicular helper T cells were higher in the low-risk group compared to the high-risk group. Meanwhile, tumor-promoting cells like naive CD4+ T cells, macrophage Mo cells, and activated mast cells were detected in high-risk rather than low-risk group patients. These findings are consistent with the role CD8+T cells (43, 44) and systemic CD4+ T cells (45, 46) play in mediating durable antitumor responses (47). CD8+ T cells, especially IFNγ+ CD8+ T cells, are considered major drivers of anti-tumor immunity (48), and IFNγ could enhance the activation of naive T cells in the tumor (43). The CD4+ T cells could eliminate tumor cells directly through cytolytic activity (49).Moreover, the activated CD4+ T cells are able to reshape the tumor immune microenvironment and facilitate tumor clearance (50). So, more infiltration with CD8 T and activated CD4 T cells and less activation of naïve CD4 T cells is beneficial for HNSCC prognosis.

In addition, an obvious infiltration of activated mast cells (MCs) was observed in high-risk group. Infiltration with mast cells inside tumor microenvironment correlates with increased intratumoral microvessel density, enhancing tumor growth and tumor invasion, and inducing overall poor clinical outcome (51). Activated MCs promote IL-6 expression and decrease Th1/Th2 cytokines to skew Tregs towards IL-17-producing T cells (Th17) (52). These results suggested that reduced Treg cell recruitment with concominant increase in Th17 infiltration may affect the poor prognosis (53, 54).

M0 macrophages accounted for a higher proportion of high-risk group, indicating that they may also play an immunosuppressive role inside tumor immune microenvironment (55). However, we didn’t observe statistically significant differences of M1 and M2 macrophage populations between low- and high-risk groups. Since the infiltration counts of M1 and M2 macrophages were unknown, we predict that tumor-associated macrophage polarization (M1/M2 ratio) was another potential indicator of patient prognosis (56, 57). The overall level of Treg cell infiltration was significantly higher in the low-risk group than high-risk group.

It is generally accepted that Treg cells help promote tumor immune escape, however more recent studies suggest that Tregs are heterogeneous in nature with different Treg populations having opposite effect on tumor microenvironment (7). Two types of Treg cells were identified including suppressive resting and activated Tregs, and nonsuppressive Treg (7). In addition, studies have suggested that infiltrating and circulating Treg cells may lead to different prognostic outcomes in tumors (58, 59), Taken together, quantitative understanding of how to alter microenvironment of HNSCC will come from quantifying the ratios of suppressive to nonsuppressive Treg populations. Our results confirmed that tumor immune microenvironment in the low-risk group was more beneficial for tumor killing. We therefore suggest that 17-lncRNA signature is a good measure of tumor immune microenvironment, and thus a good predictor of HNSCC prognosis.

Our study integrated bioinformatics analysis with the knowledge of tumor immune microenvironment to identify and validate the 17-lncRNA signature for HNSCC prognosis, providing a new approach for further stratification among HNSCC patients. Future studies should attempt to clarify the mechanisms of how 17 Treg-related lncRNAs regulates tumor immune microenvironment in HNSCC, and provide new targets for HNSCC immunotherapy in the future.

## Data Availability Statement

The datasets presented in this study can be found in online repositories. The names of the repository/repositories and accession number(s) can be found in the article/**Supplementary Material**.

## Author Contributions

XS designed and directed the study. QS and YL organized the public data and wrote the manuscript. QS, XY, and XW performed experimental work and analyzed the data. QS, YL, and ZL took charge for data visualization. XS, YL, and YM revised the manuscript. All authors contributed to the article and approved the submitted version.

## Funding

This work was supported by the Taishan Scholars Project (No. ts20190991) and the Natural Science Foundation of Shandong Province (No.: ZR2019PH113).

## Conflict of Interest

The authors declare that the research was conducted in the absence of any commercial or financial relationships that could be construed as a potential conflict of interest.

## Publisher’s Note

All claims expressed in this article are solely those of the authors and do not necessarily represent those of their affiliated organizations, or those of the publisher, the editors and the reviewers. Any product that may be evaluated in this article, or claim that may be made by its manufacturer, is not guaranteed or endorsed by the publisher.
