# Supplementary figures and images for: Identification and Validation of 17-lncRNA Related to Regulatory T Cell Heterogeneity as a Prognostic Signature for Head and Neck Squamous Cell Carcinoma
Source: Front Immunol. 2021 Nov 22;12:782216. doi: 10.3389/fimmu.2021.782216 (PMC8645855; doi:10.3389/fimmu.2021.782216)

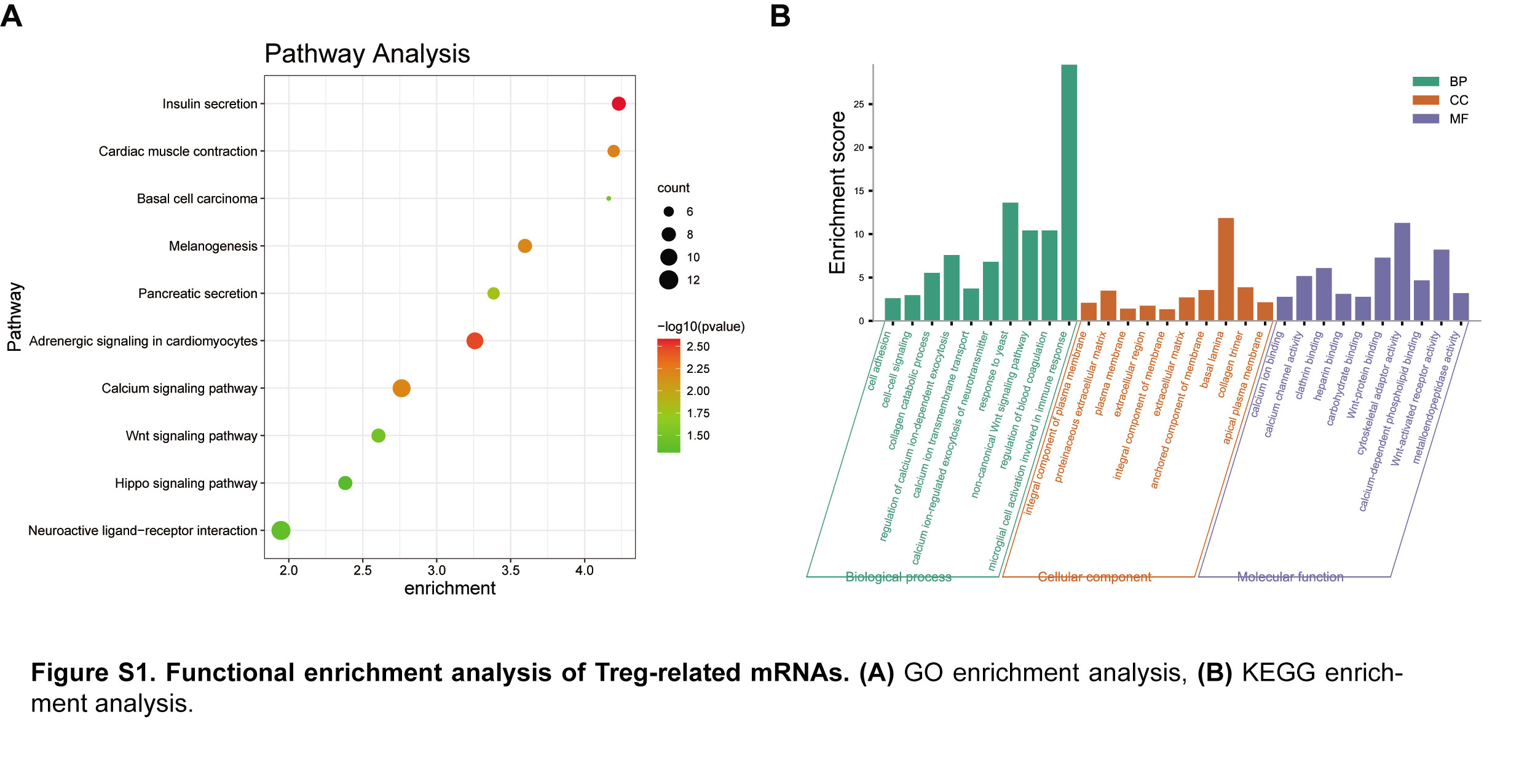

Supplement: Supplementary file 7 [file Image_1.jpeg]

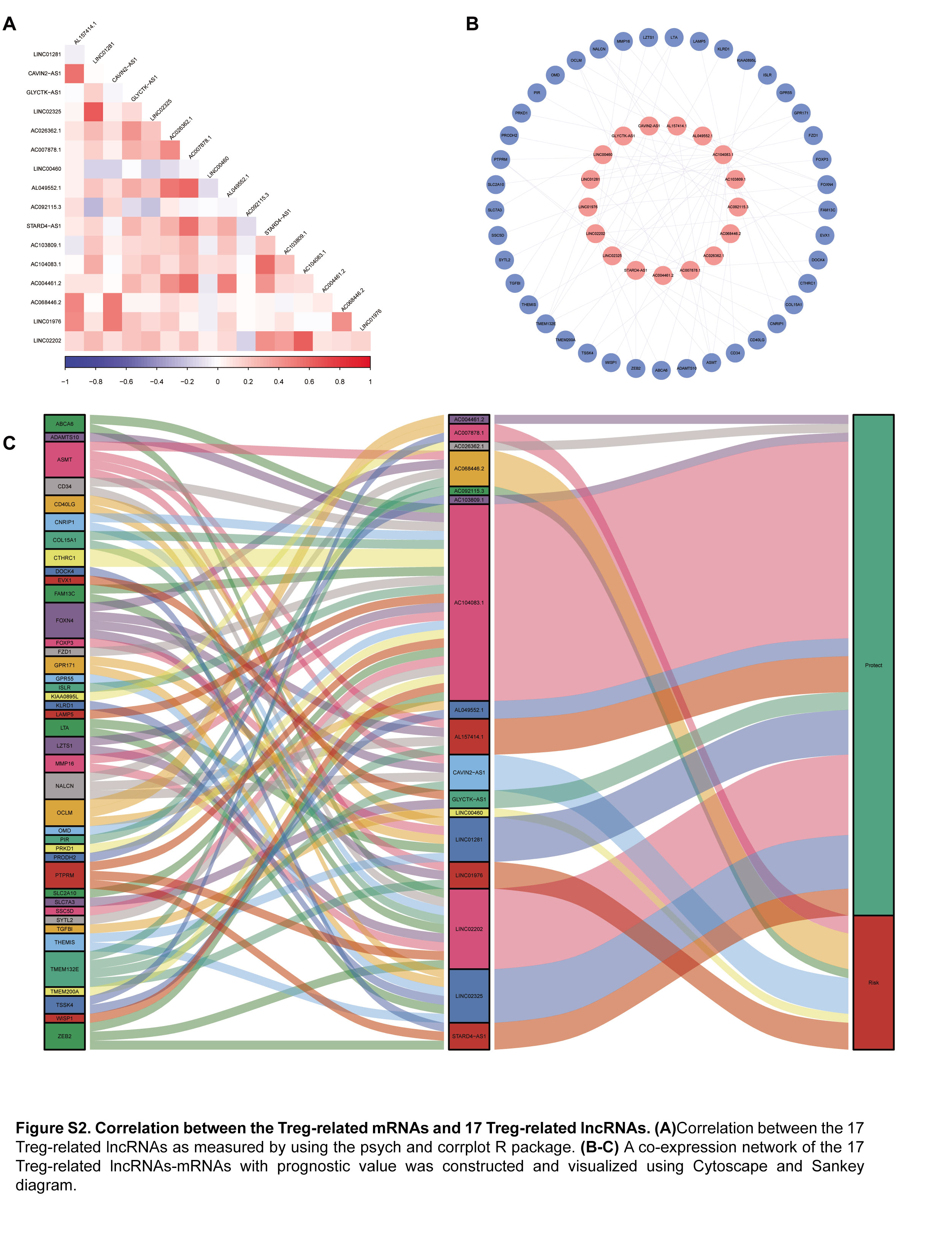

Supplement: Supplementary file 8 [file Image_2.jpeg]

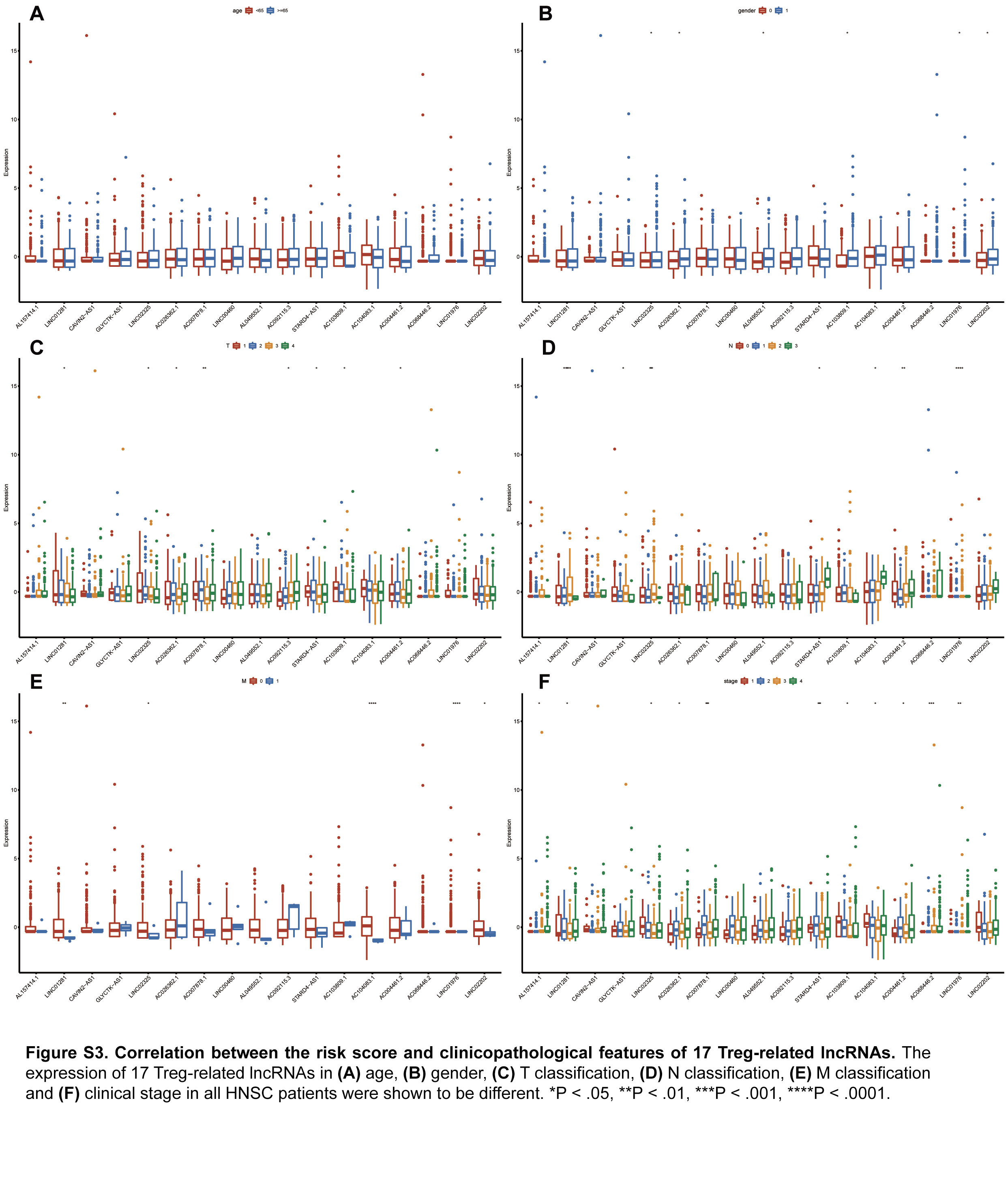

Supplement: Supplementary file 9 [file Image_3.jpeg]

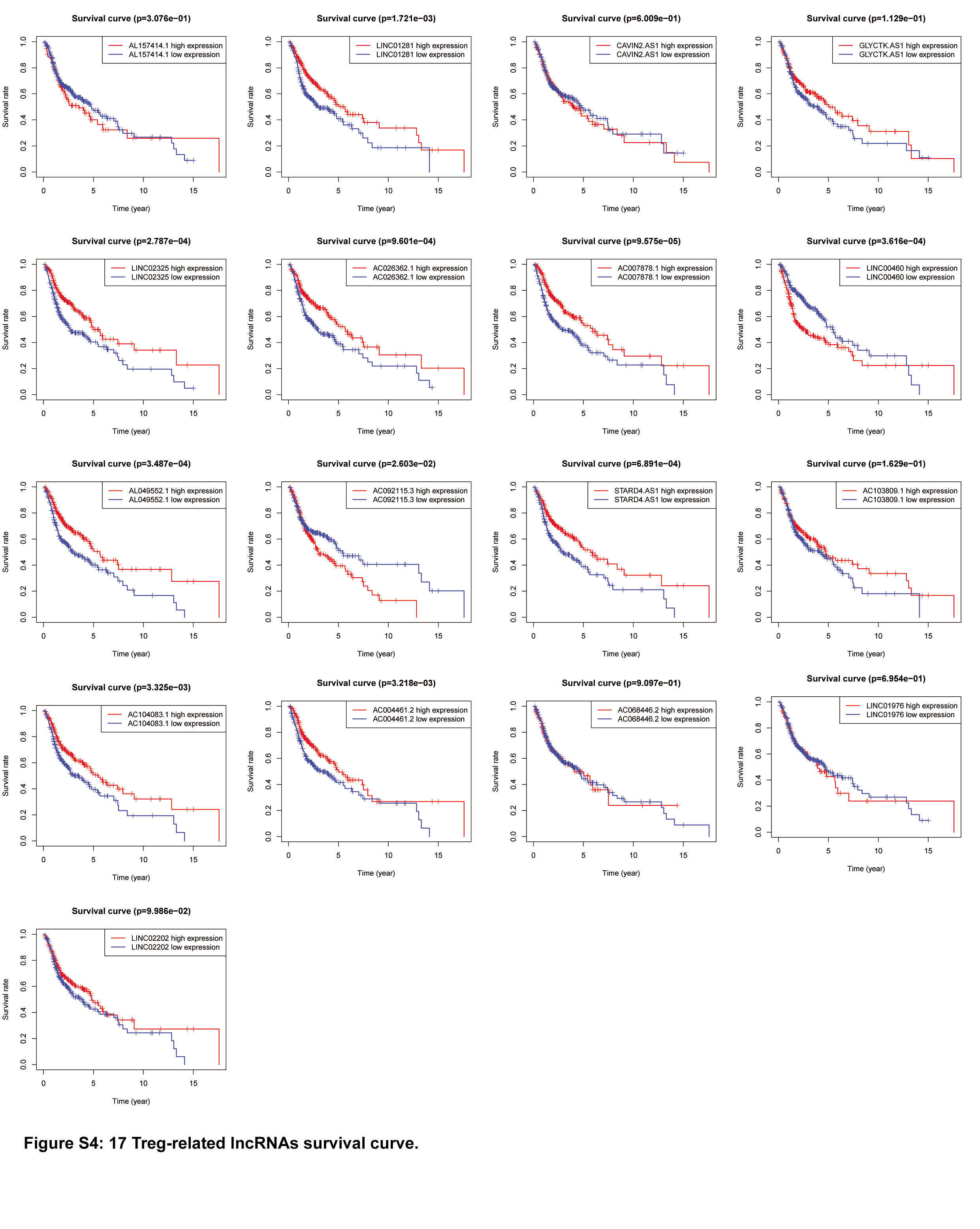

Supplement: Supplementary file 10 [file Image_4.jpeg]
